# Strength Tests of Selected Ropes Used in Mining Shaft Hoists After Their Replacement in Stochastic Interpretation

**DOI:** 10.3390/ma18174217

**Published:** 2025-09-08

**Authors:** Andrzej Tytko, Grzegorz Olszyna, Tomasz Rokita, Krzysztof Skrzypkowski

**Affiliations:** 1 Faculty of Mechanical Engineering and Robotics, AGH University of Krakow, Mickiewicza 30 Av., 30-059 Kraków, Poland; tytko@agh.edu.pl (A.T.); rokitom@agh.edu.pl (T.R.); 2 Faculty of Civil Engineering and Resource Management, AGH University of Krakow, Mickiewicza 30 Av., 30-059 Kraków, Poland

**Keywords:** steel wire ropes, safety, durability, wear, random processes

## Abstract

As the reserves of these raw materials continue to dwindle, their extraction is becoming increasingly difficult, with shaft depth increasing and sometimes exceeding three kilometres. As shaft depths increase, the costs, as well as the risks of mining and other shaft operations, increase non-linearly. There is also a significant increase in the costs associated with condition assessment, which depend on the inspection and testing method used and increase with the lifetime of the facility. New technical and organisational solutions are emerging to meet these requirements. This paper addresses the operation of steel ropes. This article analyses the results of strength tests on two selected modern hoisting rope designs that have recently come into service. These structures are relatively unknown to users in terms of their wear. In their operation, significant problems of condition assessment and safety, as well as disqualification due to the level of wear reached, arise. Strength tests were performed using classic non-destructive methods (tensile test, torsion test, bending test) to assess the technical condition of ropes after their replacement. The tests on two rope structures carried out before and after they were put down by expert decision were analyzed. The results of these tests were statistically processed and presented graphically to determine similarities and differences. Statistical analyses were used to evaluate the results by examining the distribution of variable strength parameters. All results were commented on, and specific and general conclusions were drawn. The article presents the conclusions, the most important of which is that new and complex ropes exhibit varying degrees of wear across the layers. This is due to their compaction process. These should be useful to users of similar rope designs, personnel carrying out the obligatory tests imposed by the legislation, and those making strategic decisions regarding the operation of entire mining plants. The analyses may contribute to the subsequent assessment of the technical condition of new ropes, which in many cases have wear parameters (corrosion, strength loss, etc.) assessed in a subjective, not quantitative, manner.

## 1. Introduction

Steel ropes operating in a wide range of transport equipment are valued for their special properties in the form of their ability to carry variable dynamic longitudinal loads, their remarkable flexibility giving them the ability to be repeatedly bent on mating wheels and shoots, their relatively high durability and the possibility of non-invasive assessment of wear during operation [1]. The operating conditions of steel structures pose practical design and operational problems that are of interest to engineers and researchers [2]. A negative but persistent feature is the limited fatigue life resulting from being subjected to physical wear processes due to fatigue of the wire material, susceptibility to corrosion, inevitable losses due to scuffing, wiping of galvanic coatings, ageing of grease and plastic components and being subjected to various types of deformation. These processes occurring over time during operation are cumulative and random [3]. In general, they are non-linear with an exponential growth character. This makes steel ropes interchangeable elements, as their working life is much shorter than that of the components and equipment working with them. The level of rope wear at any given time determines, to a large extent, the operational safety of the entire rope device based mainly on the number of broken wires and their propagation over time [4,5]. This affects the stress in the wires, leading to faster wear. Friction does not significantly affect the local stress and strain distribution [6]. This is particularly important for applications such as the support ropes of mining shaft hoists, which are subject to particularly harsh operating conditions. In virtually all countries, including Poland, steel ropes are subject to specific operating restrictions regulated by international or local regulations. In the case of hoisting ropes for mining shaft hoists operated in Poland, the principles of their operation in terms of selection, testing and deposition are regulated in several legal documents [7] (Supplementary in [7]). All steel ropes put into service by law must be strength tested by the manufacturer and accepted by the future user. During operation, the steel ropes of mining shaft hoists are subject to periodic non-invasive tests to determine their current condition. Independent centres carry out these examinations, and their experts, based on specific regulations (Supplementary in [7]), decide on the necessity and timing of replacing the worn rope with a new one. Two types of routine testing are practiced: periodic magnetic testing and more frequent visual testing to assess the technical condition [8,9]. Putting ropes out of service provides a unique opportunity to assess their actual degree of wear and strength degradation at the time of putting them out of service, also by invasive strength methods such as destructive testing. It should be clearly emphasized that the concepts of wear and strength degradation of ropes are not the same. The level of wear on the basis of the tests carried out is quantified individually by the appraiser, whereas strength degradation is an objectively measurable parameter that can only be determined on the basis of destructive strength tests. There is a lot of confusion and misunderstanding in this area. Strength degradation, fatigue life and durability are different concepts related to the strength of materials. Strength degradation is the process of material degradation through the gradual loss of strength properties, which leads to a reduction in the load-bearing capacity of the structure and its degradation [10]. Fatigue life, expressed as the number of cycles a material can withstand, is defined as the ability of a given material to withstand loads under specific operating conditions. It is crucial in many structures exposed to variable loads, such as cables, bridges or aircraft parts [11,12,13]. Service life is related to the total operating time a given element can function safely without critical damage. It considers fatigue life and other degradation factors such as corrosion, abrasion or material ageing [14]. That is why this paper is devoted to this issue. It presents a study of two types of modern mining hoisting ropes of very similar design and operated under comparable shaft conditions. These ropes were manufactured by a reputable rope manufacturer. After these ropes were put away, as a result of the level of wear reached and the decision of the expert, an independent institution re-performed strength tests on the most worn sections taken for testing from the ropes put away. This made it possible to analyse the test results of the new ropes and the same ropes after they had been put aside, both qualitatively and quantitatively. This was possible thanks to the availability of the manufacturer’s test results [15,16] and the results of tests carried out after the ropes were put down [17]. The results of rope strength tests are most often interpreted in terms of their average values for the whole rope. In the present study, a more comprehensive approach was taken, and the results were analysed not only in terms of their average measures but also in terms of their values for individual rope components, i.e., strand layers and wire layers in strands. Such a comparison was made possible by applying statistical analysis to the strength test results for the individual wire populations. For small sample sizes to assess rope reliability, the grey model (GM) can be used to predict data [18]. Statistical analysis is most appropriate here, as the rope designs selected for testing are made up of a large number of wires. The numbers of these populations allowed the data to be analyzed using classical statistical methods [19]. On the basis of the results obtained, conclusions were drawn about the behavior of the rope designs analyzed as a whole. For the first time, it was also analyzed what the distribution of wire strength parameters looks like in individual layers of strands of new ropes and after they have been laid. It is this issue that is the essence of this paper. To the authors’ knowledge, this type of comparative study for a class of objects such as steel ropes has not been published in the literature so far. If it has, it has considered the relationship of how the wear level of a rope translates into its strength degradation for different wear levels interpreted in terms of average values for the whole rope [20]. They operated on average values aggregated for the whole rope, rather than assigned separately to its individual structural elements. It should be noted here that ropes are composite objects made of steel wires that form a higher structure, i.e., strands. In complex ropes, on the other hand, the strands are arranged in layers.

The research results are presented in the form of numerous graphs, which, in the authors’ opinion, better facilitate the study of the subject matter than a descriptive form. Nevertheless, interpretation of the presented results requires an elementary knowledge of statistical data analysis. The results can also be applied to other areas of steel rope operation, such as cableways, crane and hoisting equipment, etc. This applies particularly to modern multi-layered ropes made of many wires and strands made by compacting technology.

Although it deals with issues related to steel rope design, which belong to the field of mechanical engineering, this paper is broader. Also, it places itself in the mining and geological sciences field, as it addresses operational problems related to the safety of mining shaft hoists. Without safely operated mine shaft hoists, there are no safe deep mines. This paper is dedicated not only to the technical personnel involved in rope surveys, but also to mine management for the economic evaluation of the operation of these equipment components.

## 2. Materials and Methods

In most cases where steel ropes are used, their manufacturer is obliged to present the results of strength tests for the ropes produced, in order to prove that their workmanship, quality and durability are in accordance with the customer’s order. This rule is particularly strictly respected in the Polish mining industry, where the regulations and rules of conduct are supervised by the District Mining Office in Katowice. The situation is similar in South Africa [21], the USA and other countries, where the practice of good operation of ropes used in mine shaft hoists is regulated at the national level. It is similar in the European Union, but, for example, the conditions and rules for the operation of ropes in and hoisting and craning equipment must comply with the law established in the EU and enshrined in the so-called EU Directives and Regulations. This law does not cover steel ropes used in mine shaft hoists and is enacted separately in each European country through internal legislation.

Routine strength testing of mine shaft hoist ropes, prior to loading, requires a section of rope several meters long to be taken from the rope and suitable samples to be prepared for these tests. These tests lead to the determination of the breaking strength of individual rope wires and the determination of additional indicators relating to their fatigue life. In the course of the tests, all specified strength test results are assigned to the respective sample. They are performed according to the relevant standards [22,23,24]. Other parameters, such as the assessment of the quality of the zinc coating of the wires and the thickness of this coating, are also subject to evaluation of the condition of new ropes. In the present study, only three basic types of strength tests were analyzed, i.e., tensile test, bending test and wire torsion test.

**Figure 1:**
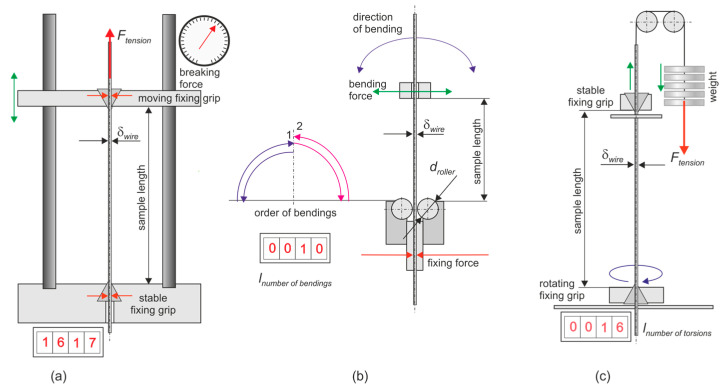
Three basic standard tests for wires: (**a**) tension, (**b**) bending and (**c**) unidirectional torsion. *Source: Author’s own work based on* [25].

Strength tests were performed according to the relevant standards. Schematic diagrams illustrating the test procedures are presented in Figure 1.

Figure 1a shows a diagram of the static tensile test according to the PN-EN-ISO-6892-1:2020-05 Metallic materials—Tensile testing—Part 1: Method of test at room temperature [22]. Sample preparation involves appropriately securing the wire in the grips of the testing machine. The samples were approximately 150 mm long. The test is carried out until the wire breaks, resulting in the tensile force at which rupture occurs. The value of the breaking force and knowledge of the wire cross-section allow for calculating the wire’s tensile strength Rm, MPa.

Figure 1b shows the biaxial bending test according to the PN-ISO 7801:1996—Metallic materials—Wire—Reverse bend test [23]. This standard specifies the wire resistance with dimensions ranging from 0.3 to 10.0 mm, and plastic deformation during a bidirectional bending test. The figure shows a bending machine used to perform this test. This test determines the plastic properties of a given wire. Test preparation involves properly mounting the sample in the machine and performing the test. The number of bends is recorded during the test until the wire completely breaks. Figure 1c shows a unidirectional twisting test using the PN-ISO 7800:1996—Wire—Simple torsion test [24]. This standard specifies the wire resistance with diameters ranging from 0.3 mm to 10.0 mm, to plastic deformation during a unidirectional twisting test. The figure shows a twisting machine used to perform this test, which determines the plastic properties of a given wire.

The results of the tests carried out by the manufacturer of the rope in question must meet the minimum strength criteria laid down in the standards. They must also be accepted by the rope user’s representative. Sometimes these tests are repeated on another rope sample at an independent testing center. This paper uses the results of tests on new ropes carried out by a rope manufacturer [14,15]. These results were verified by an independent testing center (Team of Appraisers AUTORYTET in Polkowice), which is shown in the form of notes on the original manufacturer’s test result forms. The test results of the same ropes, but after they had been decommissioned, were carried out by the same appraisal center in the same way for the ropes after they had been decommissioned and submitted to the authors [16].

## 3. Methodology and Objective of the Study, Results and Analysis

As mentioned above, all rope strength test results are assigned to a specific sample. This approach makes it possible to analyse the strength characteristics of the tested rope in terms of meeting the required parameters for the rope as a whole and at the level of its individual components. In practice, this means that strength can be examined for individual layers of wires within each strand and for the successive layers that make up the structure of these strands.

Such a detailed analytical method is critical for ropes used in deep shafts, especially modern designs incorporating polymer inserts and manufactured with classic and compact (plastically deformed) strand constructions. These advanced ropes are being increasingly implemented because of their extended service life and improved resistance to fatigue under demanding operating conditions. However, they are also associated with significantly higher costs and a more complex internal structure, including many individual wires. This complexity makes the evaluation of their wear and overall condition during operation a challenging task.

For this reason, developing a deeper understanding of such ropes’ operational behavior and durability is particularly important to both users and experts tasked with assessing their technical state. Accurate knowledge in this area directly influences the safety of mine shaft hoist operations, ensuring the reliability and proper functioning of entire mining facilities. In other words, evaluating not only the global performance but also the internal wear mechanisms of modern ropes is a key factor in maintaining economic efficiency and operational safety in contemporary mining practice.

The present study also focused on analysing the actual changes in the strength parameters of worn ropes after their discontinuation. Access to the results of strength tests on new and worn ropes has, in a way, imposed itself on the purpose and direction of the analysis. The aim of the research is to determine, for two selected rope designs, what the actual distribution of the analyzed strength parameters is in the individual structural elements, both for new ropes and for the same ropes after their discontinuation. To the authors’ knowledge, the results of such tests have not been published before, so all the results presented, and the conclusions drawn are novel. In the practice of steel rope exploitation, it is common to use average measures, which do not take into account the peculiarities of the design of the complex structures of steel ropes currently used in mining. The authors are of the opinion that the results of the research presented below, and the conclusions drawn, should increase the operational safety of modern hoisting ropes for mining shaft hoists with large drawing depths. This is possible by isolating in the design of the ropes those components of the ropes that are subject to faster wear. As a result, appraisers can gain new guidance for their appraisals.

The present study comprehensively analyzed the results obtained from a series of strength tests on selected rope designs, including new and worn variants. All procedures were performed by established standards for rope strength testing, ensuring reliability and comparability of the data. The tests encompassed the complete set of wires constituting the ropes under investigation, representing 100% of the population. Three principal tests were conducted: pull-off, bending, and one-way torsion tests.

The outcomes of these tests were subsequently assigned to the corresponding strands of both new and worn ropes, as illustrated in the figures below. In this rope type, the individual strand layers are constructed from wires of varying diameters, belonging to the same breaking strength class, Rm [MPa]. This structural feature enabled a valid comparison of test results, since the analyses were performed on the parameter Rm rather than on the absolute breaking forces of individual wires Fz [daN]. Consequently, it was possible to obtain a transparent and comparable visualization of the wear progression in the wires forming the different structural elements of the rope.

Further analyses were conducted separately for each rope component, distinguishing between outer layers, inner layers, and the core. Because identical parameters were examined for both new and worn ropes, it was possible to trace the wear evolution by comparing changes in the measured strength properties from initial installation to the withdrawal stage from service. This approach provided valuable insight into the degradation process of the rope structure.

In addition, the study highlighted that the strength parameters of more complex strands exhibited multimodal distributions, reflecting the heterogeneous behavior of wires depending on their diameter, location, and degree of exposure to operational loads. This detailed breakdown allowed for a more nuanced understanding of rope components’ mechanical performance and progressive deterioration across their service life. Methods used in these cases include those used in engineering and other sciences [26,27]. Because the analysis used statistical distributions of variables and other statistical measures, not all rope components could be subjected to such analysis. Random errors in the stochastic process are eliminated [28]. For this reason, the properties of the rope components present in samples of less than 16 were not analyzed. Classical fatigue models, including the S–N (stress–life) approach and Miner’s rule, can be extended using probabilistic formulations that incorporate Weibull-based representations of material degradation and cumulative damage [29,30,31]

Reliability analysis and durability prediction based on statistical distributions are widely described in the literature [32,33,34,35].

**Figure 2:**
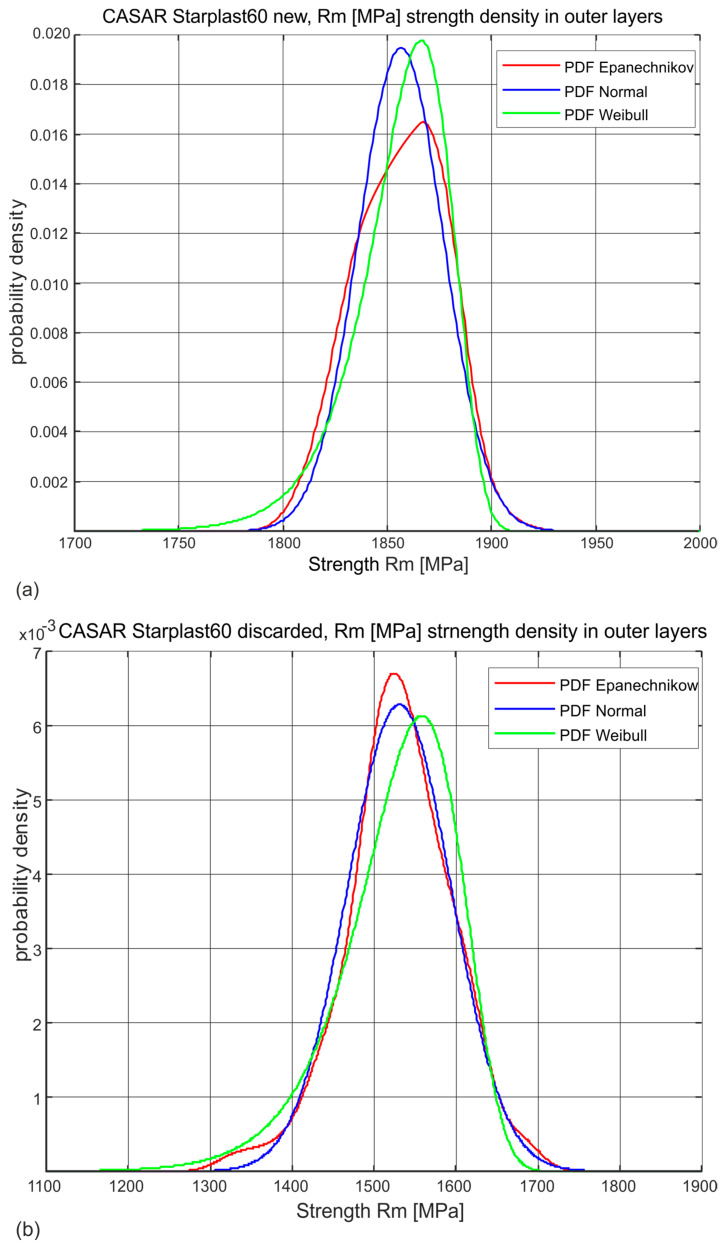
Three examples of types of estimation of probability density distributions of a selected strength variable (**a**) new rope, (**b**) rope after being discarded.

The estimation of the probability distribution of a given strength variable of the analyzed sample was adopted as the primary means of analysis. The determined histograms of the distribution of these variables allowed two types of parametric distributions of the exponential type to be adopted: the universal normal distribution and the Weibull distribution, which physically corresponds more closely to the actual distributions of these variables. A non-parametric distribution with an Epanechnikov kernel estimator was also used in the analysis of the variable Rm [MPa]. Examples of the estimation of the probabilities of one of the analyzed strength variables for new rope and worn rope are shown in Figure 2. This variable is the most represented tensile strength of the outer strand wires. The probability density function (PDF) can be derived from the lifetime curve formula [36].

In the following section, the variable Rm [MPa] for all rope wires was presented both as a non-parametric distribution with an Epanechnikov kernel and as a normal distribution. This allowed the graphical and numerical representation of the fact that the wear rate of individual rope strands in their individual layers is not uniform. The strength parameters for the individual populations of wires, derived from the individual strand layers and from the layers of these wires in the individual strands, are presented below in the form of graphs in the nature of normal distributions. The significance of such distributions for each variable was confirmed at the 95% level and did not differ from the significance determined for the same variables using Weibull distributions. However, being aware that the Weibull distribution is a right-skewed distribution, the authors considered that for the description of the parameters, the normal distribution is more useful, due to the physical interpretation of its descriptive parameters and the fact that, for the variables analyzed, it is very much shifted to the right of zero, i.e., in the right-hand limit, with a large sample size, it tends towards a normal distribution.

### 3.1. Description of Steel Rope Design Selected for Testing

**Figure 3:**
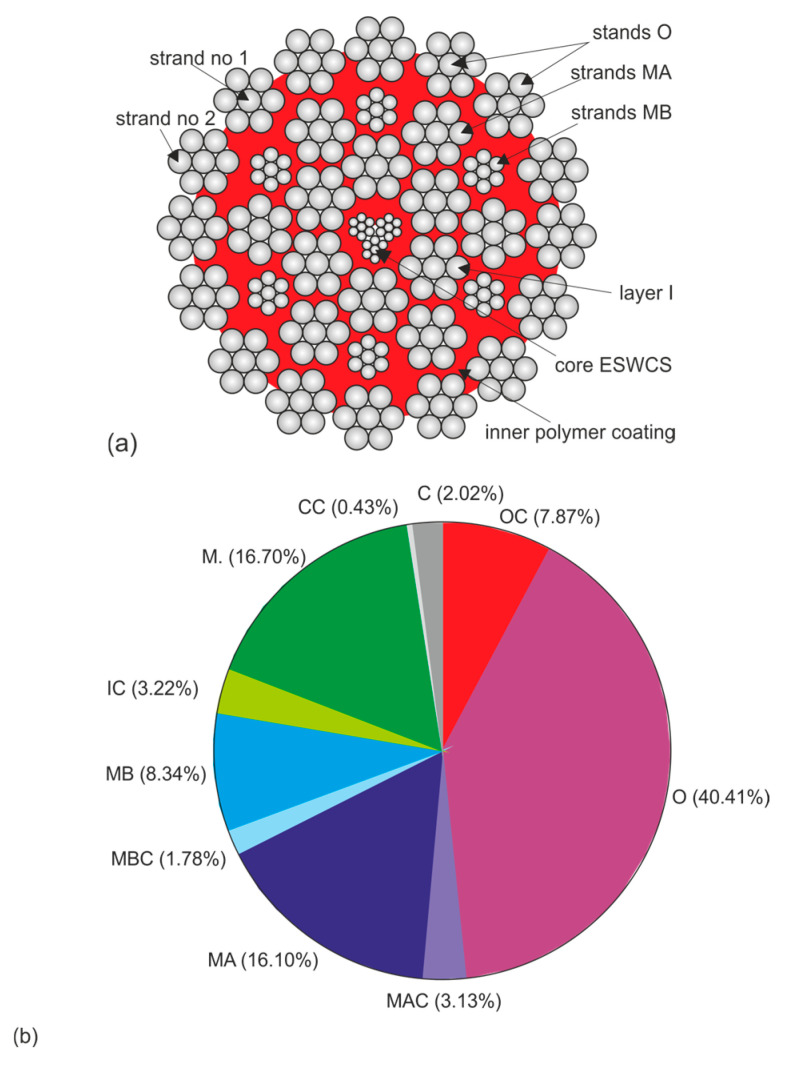
Layout of strands and wires in the STARPLAST MF 60 35(W) × 7-ESWCS rope with a diameter of 60 mm (**a**) and their proportion (**b**) in its supporting section; where: O—strands of the outer layer, OC—core wires of the outer strands, MA—strands of the larger diameter of the middle layer, MB—strands of the smaller diameter of the middle layer, I—layer of the inner strands, the letter C stands for the core wires of the strands of a given layer of strands or the core strands of the rope, ESWCS (external synthetic wire core stands)—rope core with a polymeric synthetic covering.

**Figure 4:**
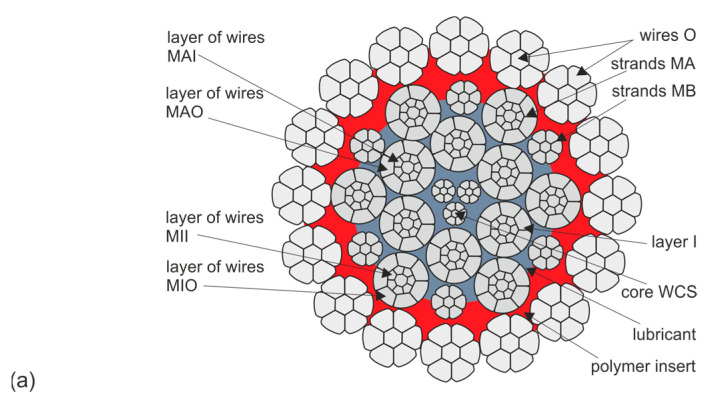

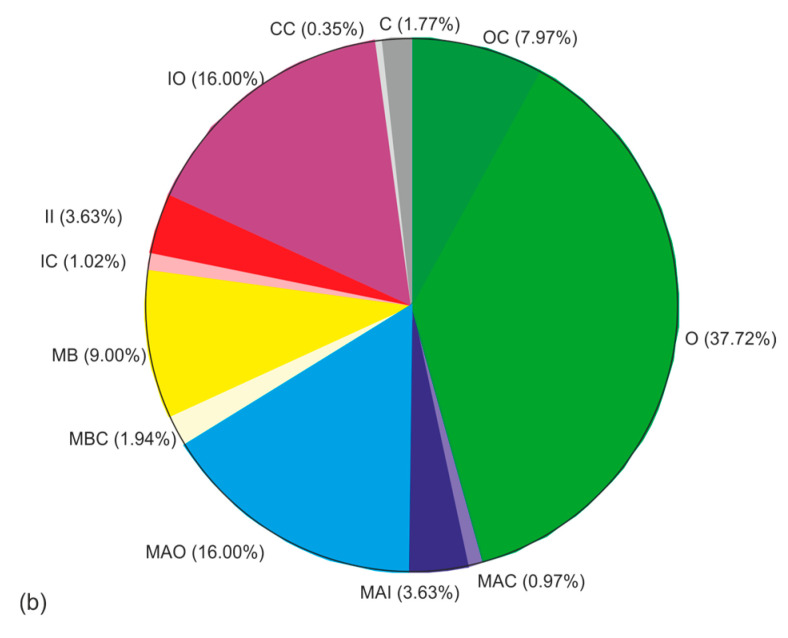
Layering arrangement of strands and wires in the STARPLAST rope with designation VMF 61 37(W) × K7-WCS 61 mm diameter (**a**) and their contribution (**b**) to its supporting cross section; where: O—outer layer strands, OC—core wires of outer strands, MA—larger diameter middle layer strands, MB—smaller diameter middle layer strands, MBC—core wires of B strands, I—inner layer strands, IC—core wires of inner I strands, WCS (Wire Core Strands)—core strands, MAO—larger diameter outer wires of middle strands; MAI—inner wires of centre strands of larger diameter; MIO—inner wires of centre strands; MII—inner wires of inner strands; C—wires of core strands, CC—core wires of core strands.

From a set of test results, made available by the supplier to the Polish mining industry of the CASAR type ropes discussed here, two types of ropes were selected for further testing. The selection criteria were threefold: similar diameter and similar design, similar working conditions of the ropes resulting in similar wear and confirmed reliability of the data on the strength parameters analyzed. The designs and basic parameters of the ropes are shown in Table 1 and Table 2, respectively, and their cross-sectional area is shown in Figure 3 and Figure 4. The parameters of the hoisting device on which the rope operated were not characterized in this study, but the conditions were very similar, and this would have added little to the analysis. Also included in Figure 3 and Figure 4 are symbols for the designation of individual wire strands and the layers of wires in the strands. These symbols continue to be used consistently in the designations in the graphs shown. Also included are pie charts showing the percentage of individual wire populations in the metallic section of a given rope. These graphs clearly show that the cross-sectional contribution of the individual wire and strand layers to the balance of the full rope cross-sectional area varies greatly. This means that even severe wear of some strands is practically irrelevant to the strength degradation of the entire rope. On the other hand, even slight wear of the strands of the outer and middle layers significantly affects the strength degradation of the whole rope. In the case of ropes with diameters of 60 mm and 61 mm, the outer strands account for almost 46% of their load-bearing section. The middle and inner layers, on the other hand, account for about 30% of the load-bearing cross-section in both ropes. Already from this comparison, it can be seen that the strength degradation of the entire rope is determined by the wear of the outer layer wires. The wear of the wires of the middle layers contributes about 30% to the total weakening of the rope, while the influence of the wear of the core layers on the strength degradation of this type of rope is negligible.

### 3.2. Analysis of Random Distributions of the Tensile Strength Rm of All Wires Taken from New and Worn Ropes

The primary purpose of the analysis carried out on all the test results of the two ropes in terms of tensile strength was to compare these designs, as they operated under similar conditions. The rope with a diameter of 60 mm was strung from strands made classically from round wires, while the rope with a diameter of 61 mm was strung from plastically deformed strands using compacting technology. Such a comparison provided an opportunity to observe the durability of ropes made from compacted strands compared to classical round strands. The second objective was to observe how wear, manifested by a decrease in the tensile strength of the individual wires, is distributed through the individual components of the rope, i.e., how the strength of the individual layers of the rope strands decreases.

**Figure 5:**
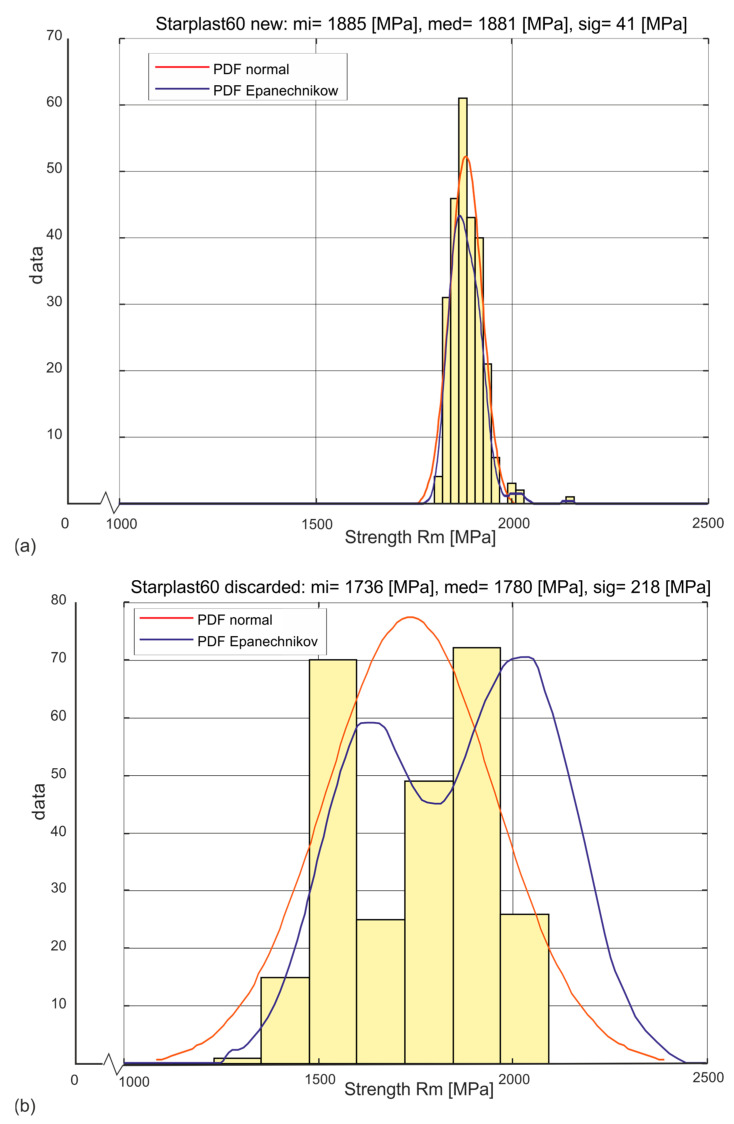
Histograms and estimated probability distributions of wire tensile strengths of a new and worn 60 mm diameter rope, where mi—mean, med—median, sig—standard deviation: (**a**) new rope, (**b**) rope after being discarded.

**Figure 6:**
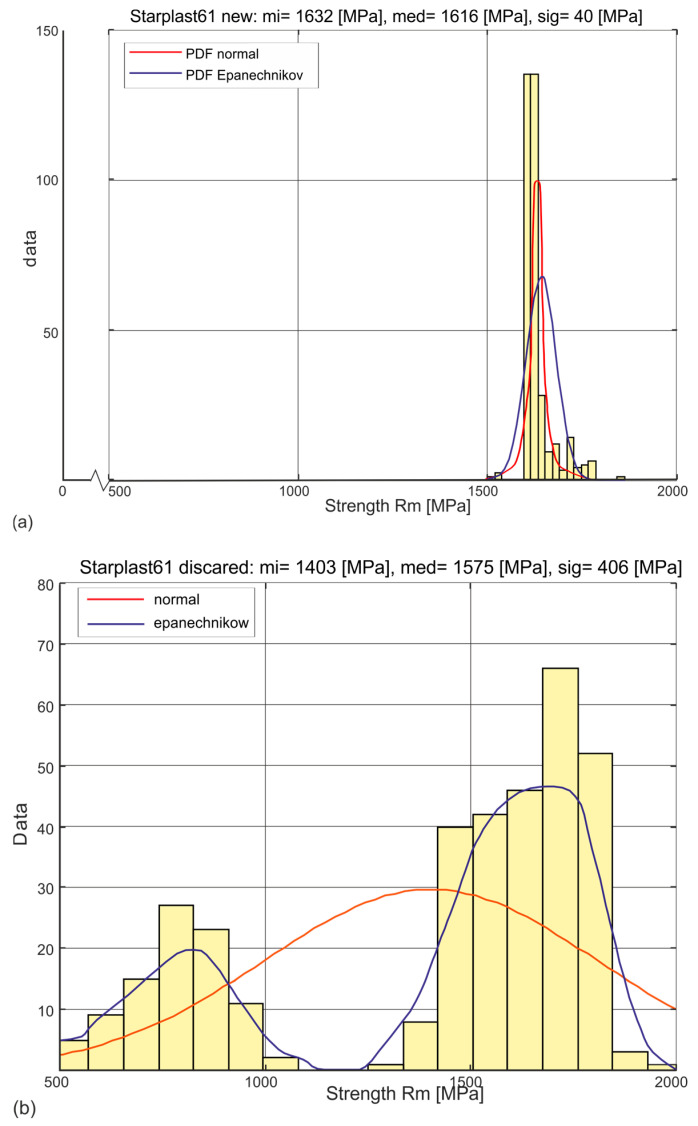
Histograms and estimated probability distributions of wire tensile strength of new and worn 61 mm diameter rope, where mi—mean, med—median, sig—standard deviation: (**a**) new rope, (**b**) rope after being discarded.

Figure 5 and Figure 6 show the histograms and estimated probability distributions of the stress distribution Rm [MPa] at which new (Figure 5a and Figure 6a) and worn rope (Figure 5b and Figure 6b) wires with diameters of 60 mm and 61 mm, respectively, broke. The histograms produced for the wires of the new ropes show a strong concentration around the mean value with small values of the standard deviations of the Rm parameter. This confirms the high performance of these wires for all diameter classes of both designs. The component wires of the compacted strands, due to the need for plastic deformation of the strands, were made in the lower strength class Rm. The histograms produced for the wires of the worn ropes show a strong concentration around two average values. One is assigned to the population of wires from strands that did not wear, and the other to the population of wires from worn strands. For both wires, this is confirmed by the ascertained non-parametric distributions with the Epanechnikov kernel estimator. They confirm that, as a result of exploitation, two populations of wires, with significantly different tensile strengths, become apparent. One population is stranded wires that are subject to wear, the other is stranded wires that are relatively not subject to wear. The identification of these populations was made by analysing the tensile strength distributions of the wires in each strand layer.

**Figure 7:**
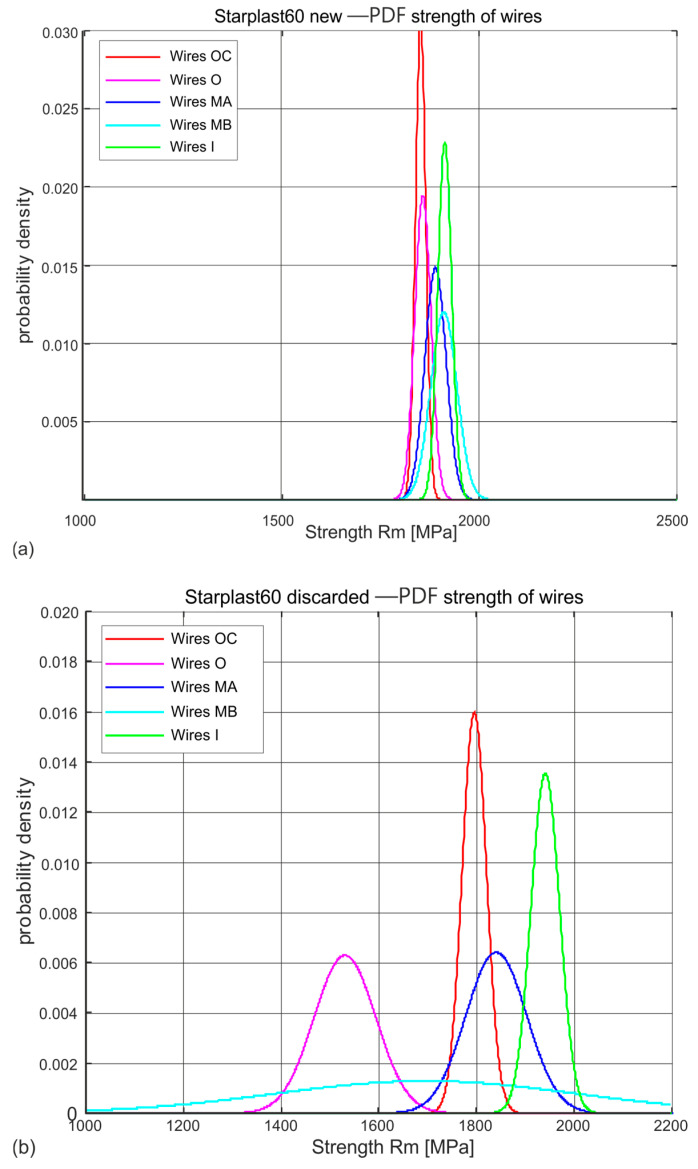
Estimated probability distributions of wire tensile strengths in individual layers of new and worn 60 mm diameter ropes: (**a**) new rope, (**b**) rope after being discarded (markings according to Figure 3).

**Figure 8:**
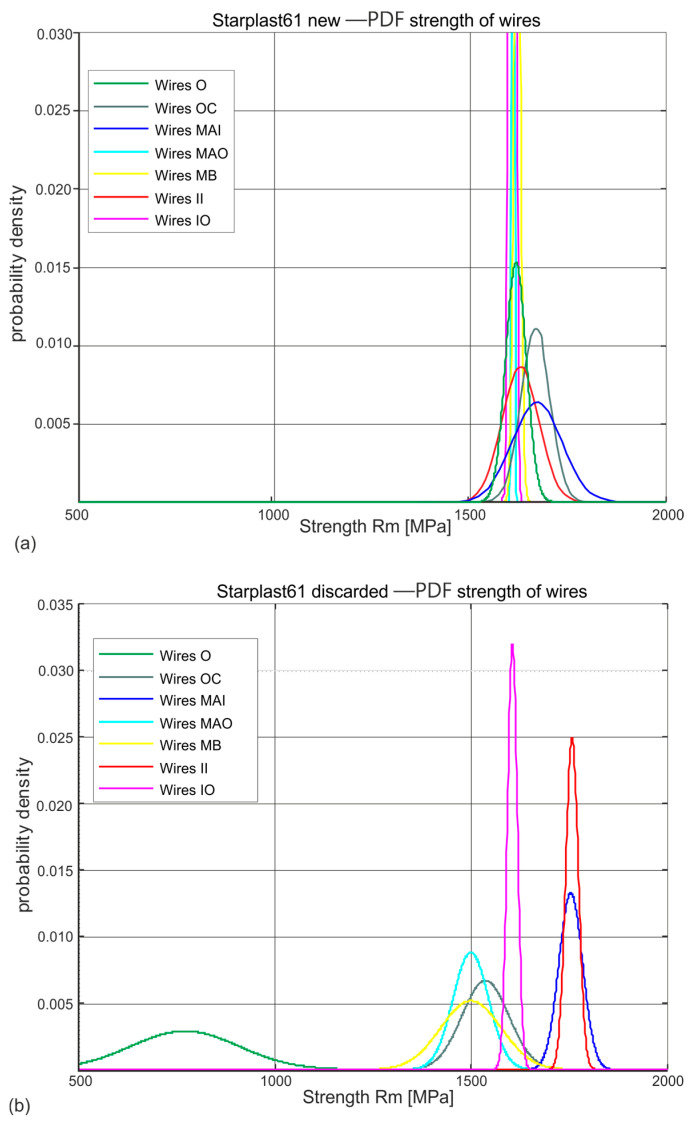
Estimated probability distributions of wire tensile strengths in individual layers of new and worn ropes of 61 mm diameter: (**a**) new rope, (**b**) rope after being discarded (markings according to Figure 4).

Figure 7 and Figure 8 show the distributions of wire tensile strengths in the different layers of strands of new and worn 60 mm and 61 mm diameter ropes, respectively.

The estimated probability distributions of the stress Rm [MPa] at which the wires of new (Figure 7a and Figure 8a) and worn (Figure 7b and Figure 8b) ropes of diameters 60 mm and 61 mm break in their respective layers, presented in Figure 7 and Figure 8, show which layers of strands are subject to fast wear, which to slower wear and which to no wear. The normal distributions ascertained on the population of new rope wires of both diameters indicate the similarity of the nature of this distribution in all the distinguished populations of strands. They also reveal a strong concentration around the mean value with small values of standard deviations. This confirms the high quality and reproducibility of the wire technology of all diameter classes. Wires taken from compacted strands, due to the necessity of performing an additional technological operation, i.e., plastic deformation of the strands, were made in a lower strength class Rm.

The Rm distributions estimated for the wire populations of the individual strands of the worn ropes indicate a strong variation in the mean values and standard deviations for each strand layer. They indicate that there is a population of very worn wires with low tensile strength. These are the wires of the outer layer strands. There are also populations of wires in which the tensile strength has decreased slightly or not at all. These are partly middle-layer strand wires and inner strand wires. In contrast, virtually no change in tensile strength was observed in the core strand wire population. The identification of these populations was made by analysing the wire tensile strength distributions of the individual strand layers. A surprise is the low tensile strength of the core wires of the inner layers. When analysing the results obtained, this reason was not identified and is most probably to be found in the technology used to make the strands of the ropes.

### 3.3. Analysis of the Number of Bends and Twists in Individual Rope Strand Layers and in the Wire Layers of These Strands for New and Worn Ropes

The section on norm tests of this paper discusses the pre-service testing of steel ropes in mine shaft hoists. It outlines the subject standardized method of carrying out bending and torsion tests on wires. Both types of these tests are used to evaluate new ropes from the point of view of the ability of individual wires to carry variable loads. The fatigue life of steel ropes is limited as they are subjected to fluctuating loads during operation, and these have the effect of reducing it. Wire rope fatigue life is calculated using the linear cumulative fatigue damage theory [37]. Many methods and fatigue damage mechanisms exist to evaluate fatigue life in many fields [38,39,40,41,42,43]. Neglecting the relationship between fatigue life and reliability may cause serviceability problems during service [44]. The bending test involves determining, under controlled conditions, the number of times a wire is bent until it fails. The results of this type of test are subject to some imperfection, due to the very short length of wire over which the bending is carried out. If notches occur in this section (e.g., from corrosion), this significantly underestimates the test results. The torsion test involves determining, under controlled conditions, the number of times the wire is twisted until it breaks. The results of this test, due to the long length of the wire sample on which the bending is performed, are more reliable and, in practice, are used to assess the fatigue life of the tested rope. The finite element method can also be used as an auxiliary tool for predicting fatigue life based on tests [31].

This paper reports and discusses the results obtained in both wire fatigue tests of selected ropes. These results have been presented in the form of the normalized normal probability distributions. The normality of these distributions was verified by appropriate statistical tests at a confidence level of 95%.

**Figure 9:**
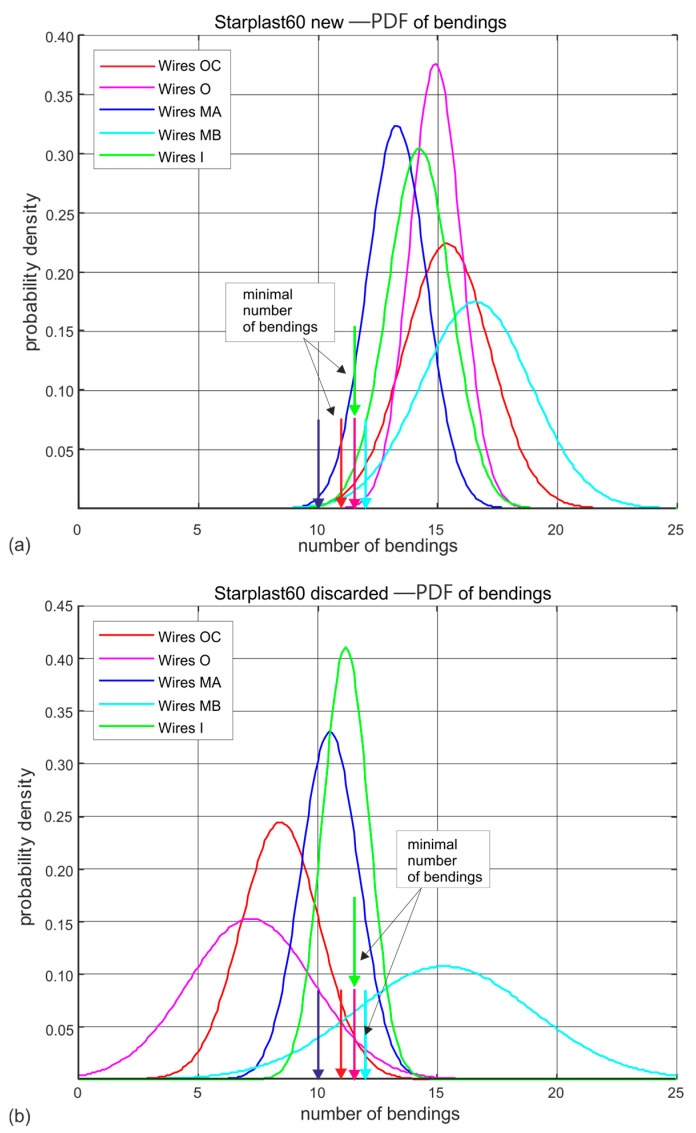
Estimated probability distributions of the number of wire bendings in individual layers of new and worn rope with a diameter of 60 mm: (**a**) new rope, (**b**) rope after being discarded (markings according to Figure 3).

**Figure 10:**
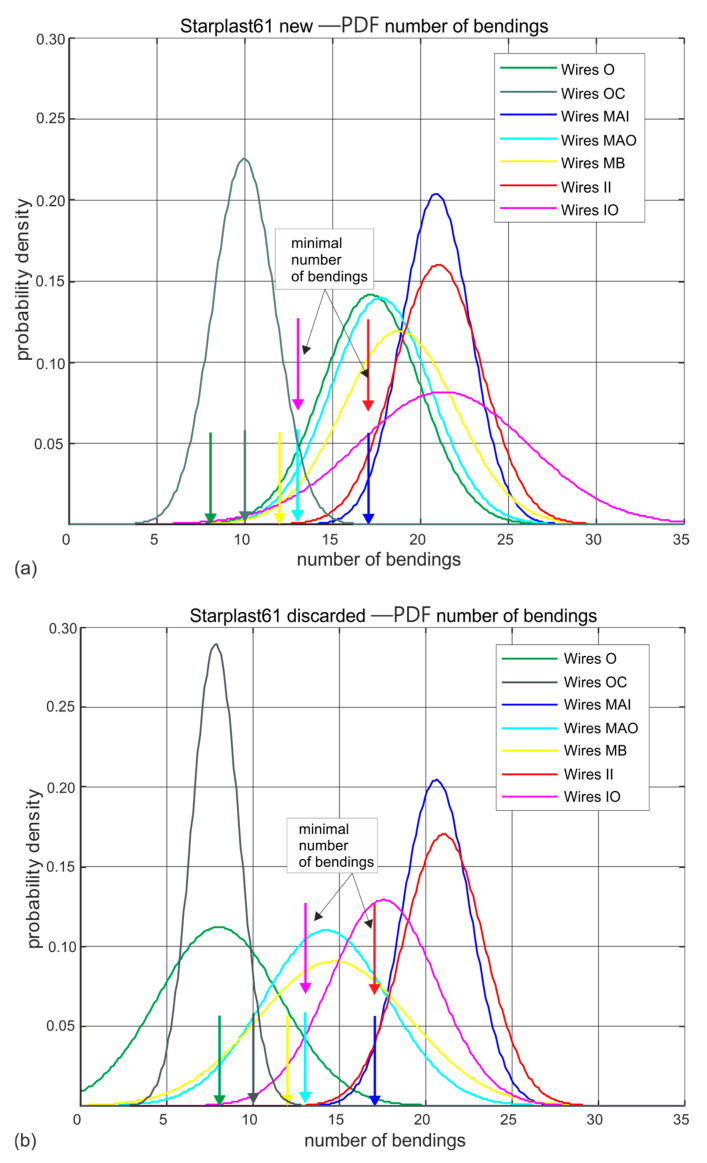
Estimated probability distributions of the number of wire hinges in individual layers of new and worn rope of 61 mm diameter: (**a**) new rope, (**b**) rope after being discarded (markings according to Figure 4).

Figure 9 and Figure 10 show the estimated probability distributions of the number of hinges at which the wires located in the individual layers of strands of ropes 60 mm and 61 mm in diameter broke for new (Figure 9a and Figure 10a) and worn (Figure 9b and Figure 10b) ropes. Presented, they show which strands of the rope wear more quickly and which wear to a negligible extent. These drawings additionally show by vertical arrows the nominal (standard) permissible number of bends that wires of a given diameter should withstand. This allows easy interpretation of the results shown. This visualization also confirms the very good wire quality of all diameters of both rope designs. The only anomaly observed is a reduction in the strength of the core wires of the compact outer strands of the 61 mm diameter rope.

Analysing the results of wire bending tests on worn ropes clearly shows a large decrease in the ductile properties of the outer strand wires, less for the middle strand wires and statistically insignificant changes in the strength of the inner and core strand wires. This is particularly evident for the 61 mm diameter rope made of compact strands. It should be noted, however, that its strength degradation at the time of laying was more than twice as high as that of the 60 mm diameter rope, whose wires retained a higher residual fatigue life in bending due to less wear at the time of testing.

**Figure 11:**
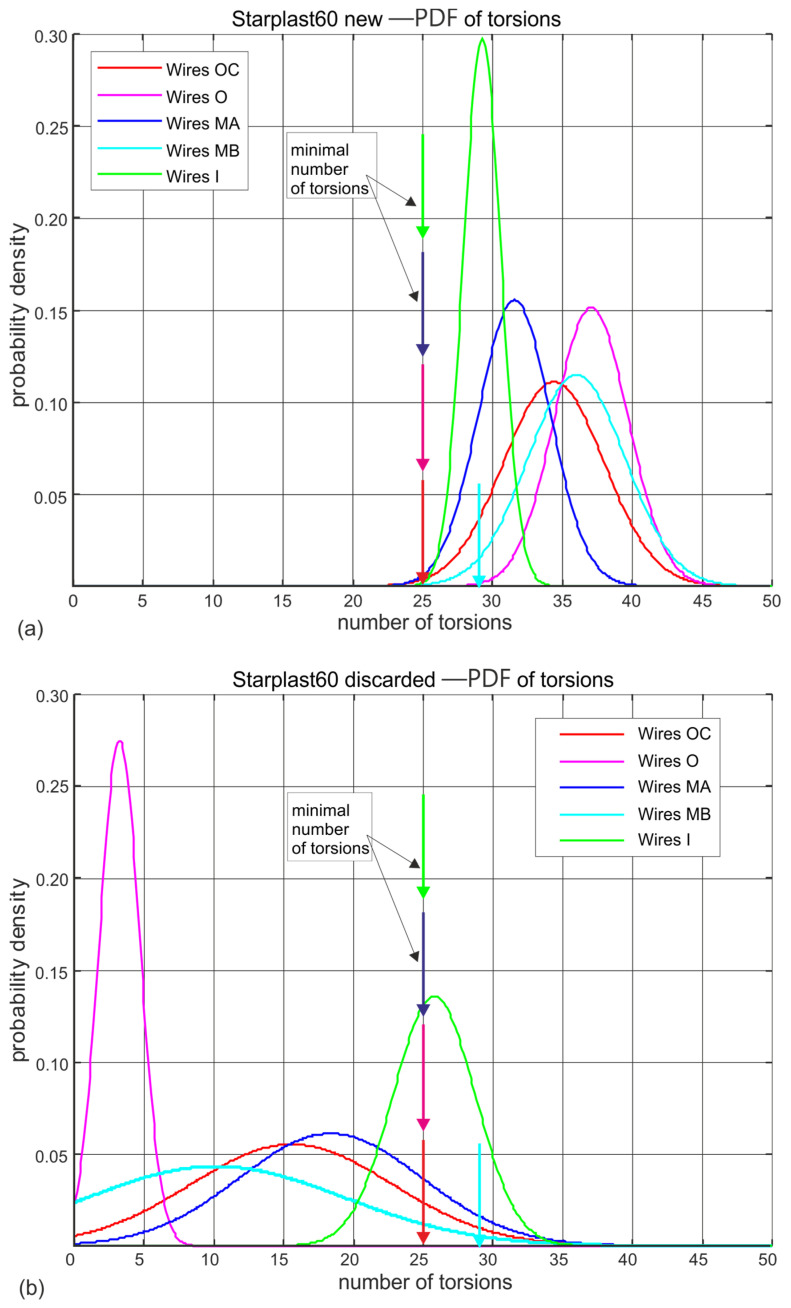
Estimated probability distributions of the number of wire twists in individual layers of new and worn rope of 61 mm diameter: (**a**) new rope, (**b**) rope after being discarded (markings according to Figure 3).

**Figure 12:**
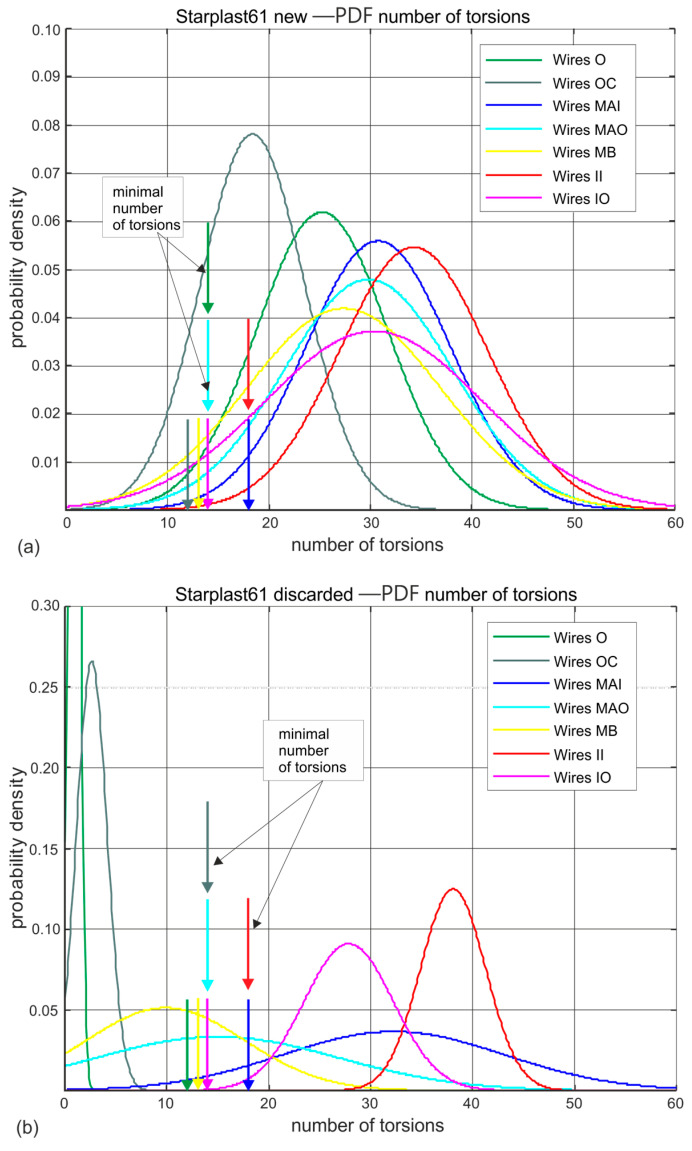
Estimated probability distributions for the distribution of the number of wire twists in the individual layers of new and worn 61 mm diameter rope: (**a**) new rope, (**b**) rope after being discarded (designations according to Figure 4).

Figure 11 and Figure 12 show the estimated probability distributions of the number of turns at which the wires located in the individual strand layers of 60 mm and 61 mm new (Figure 11a and Figure 12a) and worn (Figure 11b and Figure 12b) ropes broke. They show which strands and layers of wires wear faster and which do not. In these drawings, vertical arrows additionally indicate the nominal permissible (standard) number of turns that wires of a given diameter should withstand. This visualization allows easy interpretation of the results presented and confirms the very good workmanship and high fatigue life of the wires of all diameters of both rope designs. The wire twisting test confirms the anomaly observed, that there is a noticeable reduction in the strength of the core wires of the compact strands of the outer layer of the rope diameter of 61 mm. It is not known whether this applies to all the strand core wires of the other layers, as due to the small sample size, such an analysis for the less numerous strands was not performed.

Analysing the results of the wire torsional strength tests on worn ropes clearly shows a very large decrease in the ductile properties of the outer strand wires, a smaller decrease in the middle strand wires and statistically insignificant changes in the strength of the inner and core strand wires. This is evident for both ropes, despite the fact that the strength degradation of the 61 mm diameter rope at the time of deposition was more than twice that of the 60 mm diameter rope. The results of the wire torsional strength tests indicate that for both ropes analyzed, the outer and core strand wires have virtually lost their torsional resistance.

This study analyzed modern mining ropes’ strength characteristics, focusing on new and used rope samples. The study enabled a detailed analysis of wear and degradation throughout the complex rope structure by applying destructive testing results to specific strands and wire layers. Testing included evaluation of breaking strength, bending strength, and torsional strength, and the results were analyzed in terms of ultimate strength (Rm) to allow for straightforward comparison. The analysis revealed that different components, particularly in ropes with polymer inserts and plastically deformed strands, wear at different rates, and more complex strands exhibit a multimodal strength distribution. These findings are crucial for improving operational safety, guiding rope evaluation, and optimizing rope design to enhance durability and performance in deep-shaft mining operations.

## 4. Conclusions

The safety and reliability of mine shaft hoisting systems depend critically on the operational condition of steel wire ropes. Due to their complex structure and exposure to severe mechanical and environmental stresses, these ropes undergo progressive degradation that directly influences their fatigue life and serviceability. For this reason, a detailed investigation of the wear mechanisms, strength parameters, and degradation patterns of different rope constructions is essential for improving design and guiding statutory inspection procedures.

This study presents the results of comparative analyses of CASAR-type ropes made of compacted strands and ropes constructed from traditional round wires. The findings provide valuable insights into the mechanisms that govern rope performance in mining shaft hoists by examining the distribution of wire strength parameters, layer-specific wear behavior, and the relationship between rope cross-sectional design and fatigue degradation. The conclusions also highlight the practical implications for inspection and assessment methods, including the importance of outer layer wires, the reliability of statutory strength degradation limits, and the role of operating conditions in rope fatigue. The key conclusions are summarized below:

1. The investigated rope, made of strands using compacting technology, shows a higher fatigue life than a rope made of strands traditionally made of round wires, but the characteristics of the distributions of the strength parameters of the compacted strand wires, despite their high quality, are more variable. This is probably due to the new random variable that is introduced into the wires by the additional surface stresses during the plastic deformation of the strands.
2. Strength tests on the wires of the individual strand layers of both selected CASAR-type rope designs confirm very different rates of wear. The fastest and greatest wear is experienced by the wires of the strands of the outer layers, with slightly slower wear of the strands of the middle and inner layers. Practically no wear is recorded on the core layers of the rope.
3. In the rope designs studied, the share of the cross-sectional area of the outer layer strand wires in the total cross-sectional area of the rope is the greatest. It is the wear of the strand wires of this layer that has the greatest impact on the strength degradation of the ropes in question. This is an important observation and an important indication for experts carrying out the obligatory statutory tests of this type of rope.
4. The analyzed case of the 61 mm diameter rope, made of compact strands, confirms that the criterion for the deposition of hoisting ropes of mining shaft hoists, defined as a permissible strength degradation of 20% should not be exceeded in any case. Strength tests on the wires of the outer, middle and, to a lesser extent, inner strand layers indicate that their fatigue life is almost completely exhausted.
5. The analyzed case of a 60 mm diameter rope, made of strands of round wires, and disqualified due to the occurrence of corrosion changes visible on the surface of the wires and deformation (floating out) of the interstrand polymer filling, indicates that this rope was prematurely put down. It should be noted here that, apart from the magnetic test results in the form of regular noise recorded on the channels of LD-type inductive sensors, the appraiser has no more precise indicators of wear. He must therefore rely on experience, and this is best gained by carrying out strength tests on the wires of these ropes after the ropes have been put down.
6. The results of tests carried out on selected CASAR rope structures and presented in this analysis show that even when these ropes reach the permitted 20% strength degradation regulations, they do not cause degradation of the core layers and full degradation of the inner strand layers. The condition of the ropes is determined by the wires of the outer layers, and these are also assessable by visual methods, including a 3D visual method based on scanning the rope surface with a laser camera system [45]. It should also be noted that none of the ropes tested entered the phase of accelerated wire breakage. This is particularly true of the 61 mm diameter rope, whose actual strength degradation exceeded the regulatory limit of 20%.
7. The results of tests of the tensile bending and torsional strengths of all wires of two selected similar CASAR-type rope designs and operating under similar operating and environmental conditions of mine shaft hoists confirm that operating time and conditions are the primary factor in their degradation and, consequently, the progressive decline in fatigue life.

## Figures and Tables

**Table 1:** Technical data of STARPLAST MF 60 35(W) × 7-ESWCS rope with a diameter of 60 mm [16].

Description	Parameter and Its Value
Manufacturer	CASAR Drahtseilwerk Saar GmbH Deutschland
Rope certificate number, drum number	W6 00627243, 6270602
Rope design	16 (1 × 3.35 + 6 × 3.10) + 6 (1 × 3.45 + 6 × 3.20) + 6 (1 × 2.60 + 6 × 2.30) + 6 (1 × 3.50 + 6 × 3.25) + 3 (1 × 1.80 + 6 × 1.60)
Durability Rm	1770 MPa
Metallic cross-section	1791.4 mm^2^
Design	Wright co-wound Z/z
Galvanization	Type B (BEZINAL coating—strands of the outer layer).
Mass of 1 m	15.82 kg
Grease	ELASKON II STAR
Total rope breaking load	337,576 daN
Actual force breaking the rope as a whole	254,750 daN
Endurance performance	0.754
Rope working area	KWK “Mysłowice-Wesoła”, “Piotr” shaft
Safety factors of the rope assuming	Human driving: 8.34 > 6.97 Material transport: 7.0 > 5.97
Rope working time	50 months
Reason for discontinuation	Surface corrosion visible on all wires of the 16 strands of the outer layer, plastic filling was also observed squeezed out in the grooves between the strands, lack of grease in the grooves between the strands was observed
Weakness at the time of discontinuation	7.9%—decrease in total breaking force of all wires

**Table 2:** Technical data of STARPLAST VMF 61 37(W) × K7-WCS rope with diameter of 61 mm [16].

Description	Parameter and Its Value
Manufacturer	CASAR Drahtseilwerk Saar GmbH Deutschland
Rope certificate number, drum number	W600751141 dated 14.02.2017, 701-750231-1
Rope design	16 (1 × 3.60 + 6 × 3.20) + 6 (1 × 2.05 + 7 × 1.50 + 7 × 3.15) + 6 (1 × 2.10 + 6 × 1.50) + 6 (1 × 2.1 + 7 × 1.5 + 7 × 3.15) + 3 (1 × 1.75 + 6 × 1.60)
Durability Rm	1610 MPa
Metallic cross-section	2045.48 mm^2^
Design	right co-wound Z/z
Galvanization	Type B (BEZINAL coating—strands of the outer layer)
Mass of 1 m	18.2 kg
Grease	ELASKON II STAR
Total rope breaking load	332,020 daN
Actual force breaking the rope as a whole	272,810 daN
Endurance performance	0.821
Rope working area	Mine Salt “Klodawa”, “Barbara” shaft
Safety factors of the rope assuming	Human driving: 9.72 > 7.096 Material transport: 6.99 > 6.096
Rope working time	48 months
Reason for discontinuation	Surface corrosion and numerous pitting corrosion points were visible on the outer wires of all 16 strands of this layer, plastic filling was observed squeezed out of the grooves between the strands, a lack of lubricants was observed in the grooves between the strands
Weaknesses at the time of discontinuation	20%—total rope breaking load of the entire rope 23.25%—actual force breaking the rope in ENTIRETY

## Data Availability

The original contributions presented in this study are included in the article material. Further inquiries can be directed to the corresponding authors. While CASAR provided raw data, analytical independence was maintained through third-party validation by AUTORYTET [17].
